# Dopamine in socioecological and evolutionary perspectives: implications for psychiatric disorders

**DOI:** 10.3389/fnins.2015.00219

**Published:** 2015-06-16

**Authors:** Yoshie Yamaguchi, Young-A Lee, Yukiori Goto

**Affiliations:** ^1^Section of Cognition and Learning, Department of Cognitive Science, Primate Research Institute, Kyoto UniversityInuyama, Japan; ^2^Department of Food Science and Nutrition, Catholic University of DaeguGyeongsan-Si, Korea

**Keywords:** primates, social hierarchy, social interaction, evolution, dopamine, psychiatric disorder, genetic variants

## Abstract

Dopamine (DA) transmission in brain areas such as the prefrontal cortex (PFC) and nucleus accumbens (NAcc) plays important roles in cognitive and affective function. As such, DA deficits have been implicated in a number of psychiatric disorders such as schizophrenia and attention deficit/hyperactivity disorder (ADHD). Accumulating evidence suggests that DA is also involved in social behavior of animals and humans. Although most animals organize and live in social groups, how the DA system functions in such social groups of animals, and its dysfunction causes compromises in the groups has remained less understood. Here we propose that alterations of DA signaling and associated genetic variants and behavioral phenotypes, which have been normally considered as “deficits” in investigation at an individual level, may not necessarily yield disadvantages, but even work advantageously, depending on social contexts in groups. This hypothesis could provide a novel insight into our understanding of the biological mechanisms of psychiatric disorders, and a potential explanation that disadvantageous phenotypes associated with DA deficits in psychiatric disorders have remained in humans through evolution.

## Introduction

Dopamine (DA) plays important roles in various aspects of brain function (Grace et al., 1998; Robbins, 2000) and its deficits have been implicated in a number of psychiatric disorders such as schizophrenia (Seeman, 1987; Howes and Kapur, 2009) and attention deficit/hyperactivity disorder (ADHD) (Arnsten, 2006).

In rodents and higher mammals including humans, DA neurons are located in the midbrain nuclei, the ventral tegmental area (VTA), and substantia nigra pars compacta (SNc) (Grace et al., 1998). DA neurons in the SNc projects into the dorsal striatum, consisting of the nigrostriatal pathway, whereas those in the VTA projects into the prefrontal cortex (PFC) as well as the nucleus accumbens (NAcc) and limbic structures, consisting of the mesocortical and mesolimbic pathways, respectively (Grace et al., 1998). In particular, the mesocortical and mesolimbic DA pathways play crucial roles in mediating cognitive and affective functions (Grace et al., 1998; Robbins, 2000).

DA appears to be an evolutionarily old neurotransmitter system. Use of DA molecules in neurotransmission can be found in primitive organisms such as *C. elegans* (Welsh, 1972). Moreover, genes, structures, and function of DA receptors with D1 and D2 subtypes are relatively conserved across invertebrates and vertebrates (Cardinaud et al., 1998; Mustard et al., 2005). Thus, original primary function of DA could have started with primitive ones such as motor control and reward reinforcement learning (Svensson et al., 2003; Schultz, 2013). One of the evolutionary pressures that have made the DA system utilized in higher brain functions may be associated with social environments. Humans and animals organize and live in social groups. Increasing social complexities could have driven evolution of more sophisticated brain functions (Dunbar, 2009), through which DA has consequently been utilized into the complex brain systems. Such evolutionary processes would also result in emergence of psychiatric disorders associated with DA alterations at the same time.

This article aims to provide some insights about DA function in socioecological contexts and the biological mechanisms of psychiatric disorders associated with DA dysfunction from an evolutionary perspective. In particular, DA function has been extensively investigated and understood at an individual level. On the other hand, how the DA system works in animals and humans living in natural social groups has remained less understood. We argue that DA alterations, which are also associated with psychiatric disorders and thereby normally considered as “deficits” in investigation at an individual level, may not necessarily yield disadvantages, but even work advantageously, depending on social contexts.

## Roles of DA in cognitive and affective function

DA signaling is involved various cognitive and affective functions, which depend on brain areas where DA neurons innervate. The mesocortical and mesolimbic DA projections in the PFC and ventral striatum (including the NAcc), respectively, are involved in cognitive functions such as working memory (Sawaguchi and Goldman-Rakic, 1991; Bushnell and Levin, 1993; Zahrt et al., 1997; Muller et al., 1998; Kahkonen et al., 2001; Mehta et al., 2004; Von Huben et al., 2006), behavioral flexibility (Floresco et al., 2006; Coppens et al., 2010; Klanker et al., 2013), attention regulation (Chudasama and Robbins, 2004; Von Huben et al., 2006; Pezze et al., 2007; Agnoli et al., 2013), and decision making (Kurniawan et al., 2011; Humphries et al., 2012; Guitart-Masip et al., 2014). The mesolimbic DA pathway in limbic structures such as the amygdala has also been shown to contribute to affective function such as fear conditioning (Pezze and Feldon, 2004). One such cognitive functions strongly associated with mesocortical DA signaling in the PFC is working memory. Alterations of working memory function, which are similar, if not identical, patterns, caused by pharmacological modulations of DA D1 and D2 receptors have been reported across different vertebrates from rodents (Bushnell and Levin, 1993; Zahrt et al., 1997), non-human primates (Sawaguchi and Goldman-Rakic, 1991; Von Huben et al., 2006), to humans (Muller et al., 1998; Kahkonen et al., 2001; Mehta et al., 2004). This suggests that an evolutionary origin of DA system utilization on cognitive functions is quite old, and have been conserved for a long time in various species.

Genetic variants that regulate DA transmission have been shown to impact on cognitive functions. One such example is the Valine158Methionine (Val158Met) single nucleotide polymorphism (SNP: rs4680) on the gene coding catechol-o-methyl transferase (COMT) (Egan et al., 2001). COMT is an enzyme that degrades DA, such that COMT efficiency determines DA concentrations in brain areas such as the PFC where DA is released. COMT activity in Met-allele carriers is lower, and thereby DA availability is higher, than that in Val-allele carriers. Consequently, Met-allele carriers have been shown to exhibit better cognitive performance in working memory (Goldberg et al., 2003) and behavioral flexibility (Egan et al., 2001), although recent meta-analyses have reported that the effects are quite weak (Barnett et al., 2007, 2008). In contract, the Val-allele has been suggested as a risk factor for a psychiatric disorder such as schizophrenia (Glatt et al., 2003a). Percentages of Met-allele carriers significantly varies among ethnic groups. In Caucasians, percentages of the Val/Val and Met/Met genotypes are approximately 20–25% each, respectively. In contrast, a percentage of the Met/Met genotype is lower than 5%, and a percentage of the Val/Val genotype reaches even higher than 60% in Asians (Baclig et al., 2012). Accordingly, it appears that the Val/Val genotype, which is disadvantageous in terms of cognitive functions, have been paradoxically selected in Asians people. This increased prevalence of Val-allele carriers in Asians suggests that these people had been exposed to an environment where a behavioral trait associated with lower PFC DA may specifically be favorable on reproductive success.

## DA deficits in psychiatric disorders

In accordance with important roles of DA in various cognitive and affective processes, deficits in the DA system have been implicated in a number of psychiatric disorders. For instance, schizophrenia patients exhibit cognitive dysfunction such as deficits in working memory (Lee and Park, 2005), behavioral flexibility (Goldman et al., 1991), selective attention (Barch and Carter, 1998), and recall of long-term memory (Aleman et al., 1999; Reichenberg and Harvey, 2007), which have been shown to depend on DA signaling. Studies using positron emission tomography (PET) have revealed that PFC D1 receptor availability, which indicates either receptor expression, binding of DA molecules to the receptor, or DA release itself, is altered in schizophrenia patients, although the findings are quite inconsistent, with a mixture of increase (Abi-Dargham et al., 2012; Poels et al., 2013), decrease (Okubo et al., 1997; Kahkonen et al., 2001), or no change (Karlsson et al., 2002) of D1 receptor availability. In contrast, relatively consistent findings have been reported for alterations in the mechanisms of presynaptic DA synthesis in schizophrenia (Howes and Kapur, 2009). Antipsychotic drugs for treatments of schizophrenia essentially have a DA D2 receptor antagonism (Seeman, 1987). Although postmortem and human imaging studies have hitherto been unable to find a significant alteration of D2 receptor expression in the striatum of schizophrenic brains compared to that of normal subjects (Howes and Kapur, 2009), a recent genome wide association study has identified more than 100 genetic variants associated with schizophrenia, which includes one on the DA D2 receptor gene (DRD2) (Schizophrenia Working Group of the Psychiatric Genomics, 2014). Genetic variants on other DA pathway genes associated with schizophrenia also include those encoding COMT (Shifman et al., 2002), DA D1 receptor (DRD1) (Allen et al., 2008), and monoamine oxidase A (MAOA) (Jonsson et al., 2003), although some of these findings are often controversial (Glatt et al., 2003a,b; Munafo et al., 2005).

Another example of a psychiatric disorder that involves DA deficits is ADHD, as suggested by observations that therapeutic treatments of ADHD are achieved by DA agonists such as amphetamine (Arnsten, 2006). Human imaging studies have reported alterations of DA transporter (DAT) expression in the striatum of ADHD individuals, although these studies are highly controversial, reporting a mixture of increase, decrease, or no change of expression (Fusar-Poli et al., 2012). Genetic analyses suggest an association of the DA D4 receptor gene (DRD4) exon III 7-repeat allele with an increased risk of ADHD (Lahoste et al., 1996), although a more recent meta-analysis has reported that this association is much weaker than it was previously thought (Gonon et al., 2012). An association of an increased risk of ADHD has also been suggested with 148 bp microsatellite located 18.5 kb to the 5′ end of DA D5 receptor gene (DRD5) (Li et al., 2006).

Collectively, involvements of DA deficits in some psychiatric disorders are convincing, although it has still been unclear exactly how the DA system is altered in these disorders, despite of tremendous efforts.

## Roles of DA in social function

Although the roles of DA in cognitive and affective functions have been extensively investigated, its roles in social function have been less clear. However, accumulating evidence suggests that DA is one of the key neurotransmitter systems that regulate social activity in animals and humans.

Social function in which DA signaling has been implicated can be expressed as variable forms from an individual level of behavior such as pair bonding (Aragona et al., 2006), parent-offspring attachment (Gammie et al., 2008), affiliative relationship (Cervenka et al., 2010), aggression (Couppis et al., 2008), play (Achterberg et al., 2014), social recognition and memory (Millan et al., 2007), and vocal communication (Leblois et al., 2010; Willuhn et al., 2014), and prosocial behavior (Saez et al., 2015), to a social structural level such as social network (Fowler et al., 2011) and social hierarchy (Nader et al., 2012).

Social behavior at individual level has been mostly investigated in rodents. For instances, strain difference of aggressive behavior in mice and its association with D1 and D2 receptor expression has been shown, with lower NAcc D1 receptor expression and higher aggression in an inbred strain of BALB/c mice compared to another inbred strain of A/J mice that exhibit higher NAcc D1 receptor expression and lower aggression, and vice versa in D2 receptor (Couppis et al., 2008). A recent study using optogenetic manipulation of the DA system has also unveiled that ventral tegmental area (VTA)-NAcc DA transmission and activation of D1 receptor promotes social interaction with other mice (Gunaydin et al., 2014). Collectively, these studies suggest D1 receptor signaling is particularly important in the regulation of social interaction with mates. However, it is also interesting to note that this appears to be opposite in formation of pair bonding in monogamous prairie voles in which NAcc D1 receptor has been shown to prevent, whereas D2 receptor facilitates formation (Aragona et al., 2006), suggesting that involvements of DA transmission in social function could differ depending on which aspects of social behavior it mediates.

### Roles of DA in social hierarchy

DA is utilized in non-vertebrates such as ants. Recent studies have shown that DA plays an important role in social hierarchy of ants, with DA concentration significantly higher in socially dominants than subordinates (Penick et al., 2014; Okada et al., 2015). A similar observation has also been reported in a bird such as ring-necked phesants, with higher social rank males exhibiting higher striatal DA concentration (Mcintyre and Chew, 1983). In contrast, in coturnix quails, this appears to be the opposite, with higher social rank subjects determined by pecking order exhibiting lower DA concentration (Holladay and Edens, 1987). However, the mechanisms creating such difference between quails and other species are unclear.

The roles of DA signaling in social hierarchy has also been suggested in higher mammals such as rodents, non-human primates, and humans. In rodents, the roles of DA on social hierarchy has been examined in a group consisting of DA transporter (DAT) knockout mice (Rodriguiz et al., 2004). Although a group of DAT knockout mice still organizes social hierarchy as does a group of normal mice, the social hierarchy in the group of DAT knockout mice was found more unstable with frequent changes of ranking over time than that of normal mice, suggesting that appropriate DA signaling is required for organizing and maintaining social hierarchy. In relation to this finding, associations of two genetic variants on the 5′untrascribed region (UTR) of the DAT gene with social ranks have also been reported in rhesus and cynomolgous monkeys (Miller-Butterworth et al., 2008).

Studies by Nader and colleagues have shown that striatal D2 receptor availability in male and female cynomolgus monkeys under single housing is not different among subjects. However, when these monkeys are housed in a social group, and once social hierarchy is established in the group, increased D2 receptor availability, indicating higher D2 receptor expression or lower DA release, has emerged in subjects with dominant status (Morgan et al., 2002; Nader et al., 2012). Consistently with the non-human primate studies, human imaging studies have also reported a correlation between striatal D2 receptor availability and social desirability, with higher D2 availability correlating with lower social affiliation and higher social dominance (Cervenka et al., 2010). Moreover, a correlation between striatal D2 receptor availability and social status, with higher D2 availability correlating higher social status, has been reported (Martinez et al., 2010).

A recent human imaging study by Plaven-Sigray and colleagues has shown that higher D1 receptor availability in the limbic striatum is associated with higher social affiliation and lower social dominance and aggression (Plaven-Sigray et al., 2014), suggesting that not only D2, but also D1 receptor signaling also plays important roles in social hierarchy. Moreover, it appears that D1 and D2 receptors yield opposite effects in terms of social structural organization, which is consistent with opposite roles of D1 and D2 receptor in aggressive behavior of BALB/c and A/J mice (Couppis et al., 2008). One question brought up by this observation is, then, whether a high (or low) D1 receptor expression may be compensated by a high (or low) D2 receptor expression. If this is the case, social behavior of high D1/high D2 animals could be equivalent with that of low D1/low D2 animals. There has been no study investigating interactive effects of D1 and D2 receptors on regulation of social behavior. However, in the study comparing D1 and D2 receptor expression in BALB/c and A/J mice (Couppis et al., 2008), an inverse relationship between D1 and D2 receptor expression has been observed, suggesting that there may be a mechanism that prevents the counteractive effect of D1 and D2 receptors with balancing expression of these DA receptors.

Although a line of evidence suggests that serotonin (5HT) plays significant roles in social function of non-human primates (Heinze et al., 1980; Raleigh et al., 1991; Kaplan et al., 2002; Riddick et al., 2009; Embree et al., 2013; Shively et al., 2014), only a few studies have yet examined the roles of DA for regulation of social behavior in non-human primates that are housed in social groups. These studies have shown that administration of the psychostimulant, amphetamine attenuates social interaction in Java and vervet monkeys, which is reversed by a D1 receptor antagonist (Ellenbroek et al., 1989; Melega et al., 2008). In addition, administration of the D2 receptor antagonist, haloperidol also decreases social interaction in rhesus monkeys (Palit et al., 1997). Based on these observations, social interactions in non-human primates that are housed in social groups appear to be promoted and attenuated by D2 and D1 receptor stimulation, respectively, which is opposite to the findings in rodents and humans. Therefore, further investigation clarifying the mechanisms that underlie this inter-species difference is awaited.

### DA pathway genetic variants in social function

In human studies, genetic variants on DA pathway genes have been reported to affect individual social behavior and social relationships in a group. The *Taq*I A (A1) allele (rs1800497) which is one of the polymorphisms associated with DA D2 receptor function, although its location is not exactly within the DRD2 gene, and the adjunct gene encoding that encodes ankyrin repeat and kinase domain containing 1 (ANKK1) gene. Large social network studies have shown a positive correlation between the DRD2/ANKK1 *Taq*I A genotypes and friendships in the network, such that individuals with the same genotypes tend to create friendships (i.e., homophily) (Fowler et al., 2011; Boardman et al., 2012). However, a similar social network study in rhesus macaques did not find an association with DA pathway gene, but with the interactive effects of 5HT transporter-liked polymorphism region (5HTTLPR) and tryptophan hydroxylase 2 (TPH2) gene variants (Brent et al., 2013), suggesting that social network organizations in humans and non-human primates may be governed by distinct, species-specific molecules.

Both heterozygous and homozygous DRD2/ANKK1 A1-allele carriers have been reported to exhibit lower striatal D2 receptor availability than non-carriers (Thompson et al., 1997; Ritchie and Noble, 2003) [but there are also studies reporting no such difference, for instance (Laruelle et al., 1998)]. In addition, meta-analyses for studies that have investigated associations between the A1-allele and psychiatric disorders have confirmed significantly higher risks of mood disorders (Zou et al., 2012) and drug addiction such as alcoholism (Munafo et al., 2007) and smoking (Munafo et al., 2009) in carriers than non-carriers. Therefore, DRD2/ANKK1 A1-allele are disadvantageous at least in the modern human society. Nevertheless, prevalence of A1-allele carriers significantly varies among ethnic groups. Although only approximately 20 and 40% of Caucasian and American Blacks, respectively, are estimated A1-allele carriers, percentages of A1-allele carriers are estimated to be more than 60–80% in Jemez- and Cheyenee-Indians, respectively (Goldman et al., 1993). Indeed, higher prevalence of substance abuse including alcoholism and nicotine dependence in American Indians than Caucasians have been shown (May, 1982; Walters et al., 2002). No reliable data is available for prevalence of major depressive disorder in this ethnic group; however, the suicide rate in American Indians is highest among other ethnic groups in USA (Olson and Wahab, 2006), which may be indirectly associated with higher prevalence of MDD in American Indians.

Associations of the DRD2 polymorphisms with increased risks of schizophrenia, but other than the A1-allele such as C957T (rs6277) (Monakhov et al., 2008), C939T (rs6275) (Allen et al., 2008), and missense Cys311Ser (rs1801028) (Glatt et al., 2003b) variants, have also been reported. Collectively, paradoxically to the suggested disadvantageous effects of the DRD2/ANKK1 A1-allele and possibly other DRD2 genetic variants, these seemingly disadvantageous genotypes could be selected under specific environments. Given the roles of D2 receptor signaling in social function, such specific environments that contribute to selections of disadvantageous DRD2 genotypes may be associated with socially-relevant ones such as social hierarchy.

## DA function and dysfunction in a socioecological perspective

Considering the above discussions, one question has emerged; whether DA alterations and associated behavioral phenotypes, which underlie psychiatric disorders, may be understood as “deficits,” especially in social contexts? This question is particularly coined by the observations that unfavorable genotypes associated with psychiatric disorders such as Val-allele of COMT and DRD2/ANKK1 A1-allele are selected in some human ethnic groups.

A selection of a genotype that results in a seemingly disadvantageous phenotype has been implicated as early as 1958 by Fisher (1958). Since then, several studies have documented such selections of the unfavorable genetic variants. One of the famous cases is a sickle-cell trait (Allison, 1964). Allison found stable prevalence of the heterozygous sickle-cell gene in African people regardless of its disadvantageous phenotype such as anemia. This is, however, associated with the fact that the sickle-cell heterozygote is at advantage against malaria infection (Beutler et al., 1955). More direct empirical evidence for natural selection of genotypes and phenotypes in response to varying environmental conditions have been shown in the fruit fly, *Drosophila*. For instances, drosophila larvae maintained in crowded density at later stage of development exhibit higher survival rate in a subsequent adult over-population environment (Borash et al., 1998). In addition, higher-than-average incidences of copy number duplicates and deletions on toxin-response genes has been reported, which indicates positive selections of these genetic mutations (Emerson et al., 2008).

### Functional outcomes determined by genotype x environment interaction

One of the mechanisms at stake when an unfavorable genotype is selected over the favorable one may involve environmentally-associated epigenetic regulation of gene expression at early development.

A functional length polymorphism of the MAOA-linked polymorphism region (MAOA-LPR) has been found in rhesus macaques, with the 7-repeat (7R) allele resulting in lower MAOA activity than the 5- and 6-repeat (5R/6R) alleles (Newman et al., 2005). Such difference of MAOA activity between the alleles is also reflected as behavioral difference, such that during food competitions and ordinary social interactions with others, male macaques grown in a normal condition (i.e., reared by biological mothers or cross-fostered) with the 7R-allele (low MAOA activity) are more aggressive than those with the 5R/6R-alleles (high MAOA activity). However, this is reversed in male macaques reared in a nursery room with limited access to peers. These nursery-reared macaques with the 7R-allele exhibit lower aggression than those with the 5R/6R-alleles.

A similar observation has also been reported in rats, in which epigenetic regulation of gene expression is suggested (Weaver et al., 2004). In this study, offsprings born from low maternal care (licking) mothers exhibited high anxiety and stress response, but when these offsprings were cross-fostered with high maternal care mothers, their anxiety and stress response is reduced. On the other hand, even if offsprings were born form high maternal care mothers, they turned to exhibit high anxiety and stress response when they were reared by low maternal care mothers. This process was mediated by removal of methylation on the Nr3C1 gene, resulting in increased glucocorticoid receptor expression in the hippocampus, upon maternal licking.

### Behavioral alterations at individual level vs. social group level

A potential selection mechanism of an unfavorable genotype may not necessarily involve alterations of gene expression by environmentally-associated epigenetic processes, but such an unfavorable genotype may also simply work better under a specific environmental condition. In relation to this argument, considerations of DA function and dysfunction under natural or semi-natural (animal) social contexts and unusual (e.g., severe stressful) environments provokes some insights. Unfortunately, most previous studies have been conducted at an individual level, and their results are interpreted in “normal” (modern human) social contexts.

For instance, that consideration of brain function and dysfunction including those associated with DA signaling in animal socioecological contexts is a promising approach is illustrated by a study investigating the effects of the N-methyl-d-asparate (NMDA) antagonist phencyclidine (PCP) in socially housed capuchin monkeys (Linn et al., 1999). Acute and chronic PCP treatments have been shown to induce behavioral alterations including cognitive and sensorimotor gating deficits that resemble to schizophrenia symptoms in humans and animals (Javitt et al., 2012). Chronic PCP administration also promotes social withdrawal in rodents (Lee et al., 2005) and non-human primates (Mao et al., 2008) when it is examined at an individual level. In contrast, chronic PCP administration even facilitates affiliative social interaction without altering aggressive behavior in socially housed non-human primates (Linn et al., 1999). Such a difference of the findings suggests that socioecological backgrounds are important for understanding of the chronic PCP effects, and that behavioral phenotypes that are thought to be deficits in one condition may not necessarily be disadvantageous in another condition.

### DA D1 receptor function in a socioecological perspective

Here we discuss DA D1 and D2 receptor function and dysfunction in a non-human primate hierarchical society.

There are very few, if any, studies that have investigated D1 receptor function in animals living in a socially natural environment. We have recently conducted an experiment to investigate the effects of chronic administration of the D1 antagonist in a Japanese macaque (*Macaca fuscata*) housed in the semi-natural social group (unpublished observation). In this study, the effects of chronic dopamine D1 antagonist administration were examined in the second ranked subject within the social group. Before drug administration, the second ranked subject did not have food access before the first ranked subject. However, after drug administration, the second ranked subject had now frequently obtained foods even before the first ranked subject accessed. Nevertheless, the subject receiving drug administration did not have increased aggressive attacks from any other subjects including the first ranked subject in the group at any experimental stages. Since the experiment was conducted in a group of monkeys, it is hard to apply conventional cognitive and sensorimotor tests in the animal to examine the effects of drug administration. However, a persistent decrease in a number of goal directed actions after drug administration was observed, suggesting that cognitive process may be disturbed by drug administration. In addition, this experiment has hitherto been conducted only in a single animal, and therefore, awaits replications in future investigation. Nevertheless, the results suggest that behavioral traits associated with low DA D1 signaling may not be interpreted as “deficits,” but rather “beneficial” in the social contexts of animals such as Japanese macaques.

This finding in Japanese macaques could be interpreted along with the recent human imaging study showing that higher D1 receptor availability, which indicates either higher receptor expression or lower DA release, in the limbic striatum is associated with higher social affiliation, as well as lower social dominance and aggression (Plaven-Sigray et al., 2014). Thus, D1 blockade may exert low affilitative as well as more dominant and aggressive behavior. These behavioral traits associated with low D1 receptor expression would yield beneficial impacts in relatively higher social ranking subjects with close power balance between them in the group. In contrast, D1 blockade in low social ranking subjects would yield adverse impacts, which may cause more frequent defeats by higher social ranking subjects.

Thus, whether low and high D1 receptor signaling could be beneficial or detrimental may be determined by social status within a group in animals such as macaques who organize and live in a hierarchical social group. Therefore, there would be an opportunity that behavioral traits associated with low D1 receptor function are selected over high D1 receptor function, depending on social contexts.

### DA D2 receptor function in a socioecological perspective

**Figure 1:**
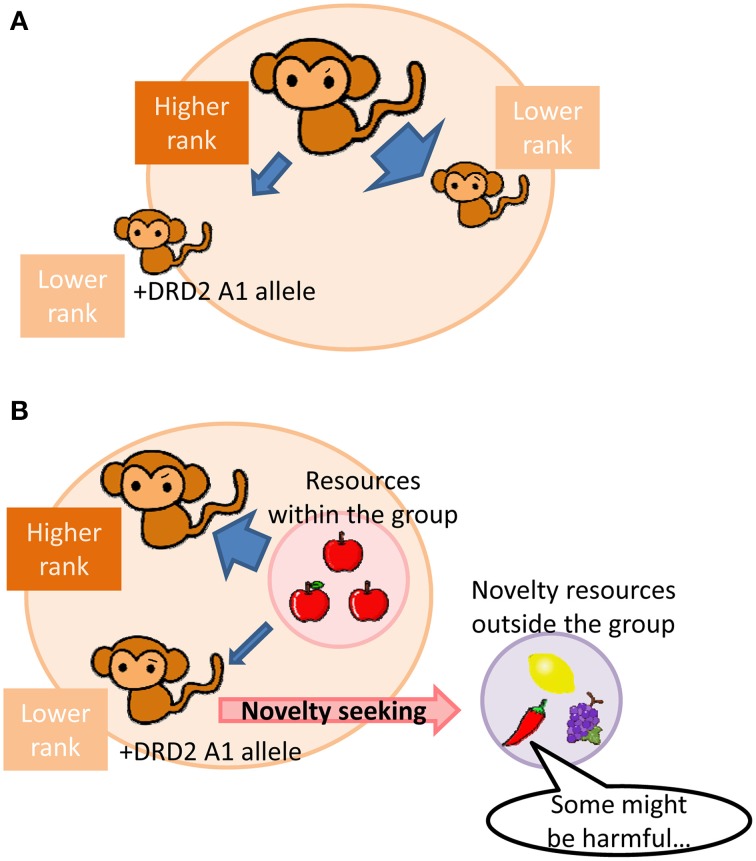
**Schematic diagrams illustrating advantageous effects of a DRD2/ANKK1 A1-allele carrier at low social status in a hierarchical group. (A)** Low social dominant and aggressive traits associated with DRD2/ANKK1 A1-allele in a lower social status subject may tend to have fewer attacks (stress) from a higher social status subject than that a non-allele carrier in a group. **(B)** A high novelty-seeking trait with the DRD2/ANKK1 A1-allele in a lower social status subject may seek a resource from outside of a group when a number of subjects within the group exceeds a maximum allowance of a resource within the group, which may in turn eventually split the group into smaller ones.

A human imaging study has shown associations between limbic striatal D2 receptor availability and social affiliation and dominance that are opposite to those of D1 receptor (Cervenka et al., 2010). Thus, beneficial effects of D2 receptor function in an animal social group are also expected to be opposite of those of D1 receptor function; low D2 receptor signaling may work advantageously in lower social ranking subjects in a group with hierarchy. Given that subjects with low D2 receptor function would exhibit a tendency of low social dominance and aggression, lower social ranking subjects with low D2 receptor function would be able to avoid attacks from higher social ranking subjects more than subjects with normal or high D2 receptor function. (Figure 1A). In contrast, subjects with low D2 receptor function would be less competitive, such that behavioral phenotypes associated with low D2 function is disadvantageous for higher social ranking subjects.

In addition to the above discussion, a number of human imaging studies have shown that D2 receptor availability in the striatum (Czoty et al., 2010; Huang et al., 2010), insular cortex (Suhara et al., 2001), and midbrain (Zald et al., 2008) are negatively correlated with novelty seeking behavior. This finding is further confirmed in animal studies in which lower D2 receptor signaling is associated with higher novelty seeking (Tournier et al., 2013). Novelty seeking could be both advantageous and disadvantageous. Novelty seeking may increase risks of life-threatening dangers in one hand, but it may also lead to new findings. Using computational model approach, Humphries and colleagues have also proposed that cortico-basal ganglia DA signaling plays significant role in this “exploration-exploitation” trade-off (Humphries et al., 2012). In a hierarchical social group, higher ranking subjects have priorities for resource access. Thus in the condition that a social group expands too large, and a number of subjects within the group exceeds more than a resource can afford, lower social ranking subjects are unable to secure the resource. A novelty seeking with low D2 receptor function would come into the advantage that aids lower social ranking subjects in the group to seek a resource from outside of the group (Figure 1B). In agreement with this idea, higher novelty seeking tendency was observed in subordinates in a cynomolgus monkey social group (Riddick et al., 2009).

Such resource seeking from outside of the belonging social group may eventually result in a split of the group. In this regards, although highly speculative, it is interesting to note that the evolutionary hypothesis of schizophrenia proposed by Stevens and Price (Stevens and Price, 2000) explains that schizophrenic traits are often observed in charismatic leaders, such that when the group gets too large, a personnel with schizophrenic traits may lead and split the group. Thus, investigation of D2 receptor function in social hierarchy and novelty seeking may eventually be able to provide a biological basis on this hypothesis.

### Selection mechanisms underlying disadvantageous genotypes

Based on the above discussion, we propose two potential mechanisms of selection of a genotype that could work advantageously in a specific adverse environment, but otherwise disadvantageous in a normal environment.

**Figure 2:**
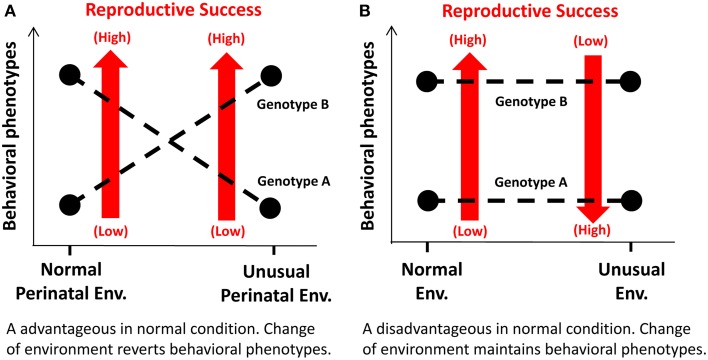
**Schematic diagrams illustrating the mechanisms underlying selection of disadvantageous genotypes over advantageous ones under specific conditions. (A)** The mechanism that involves functional alterations of a genotype through epigenetic modulation of gene expression by interacting with a perinatal environment. **(B)** The mechanism that a postnatal environment affects reproductive success of a subject in which a disadvantageous behavioral phenotype in a normal environment may work advantageously in an unusual, adverse environment.

One mechanism may involve a functional alteration of a specific genotype by interacting with an environment in early development. In this mechanism, a behavioral phenotype is reversed between specific alleles, depending on an environment that these allele carriers are exposed to early (i.e., pre- and neonatal) brain developmental periods (Figure 2A). Consequently, the allele that normally results in a disadvantageous phenotype could be favorably selected over the other allele. Such an example is illustrated in the study by Newman and colleagues that have investigated MAOA-LPR 7R-allele in non-human primates (Newman et al., 2005). Thus, macaques with low MAOA activity allele are more aggressive and competitive for food access than those with high activity allele when they are reared under normal maternal care. However, this is reversed when monkeys are reared under no or little maternal care.

The other mechanism may involve selection of a genotype under unique (and often, but not necessarily, adverse) environmental and social conditions (Figure 2B). Thus, a behavioral phenotype associated with a specific genotype may yield a disadvantage in a normal environment, but work advantageously in an unusual environment. DRD2/ANKK1 A1-allele and COMT Val-allele may be such cases.

## Psychiatric disorders associated with DA deficits from an evolutionary perspective

A recent epidemiological study has reported that fecundity rates in psychiatric patients are decreased compared to normal subjects (Power et al., 2013), which evidences that psychiatric conditions are clearly disadvantageous behavioral phenotypes in the modern human society. Nevertheless, prevalence of psychiatric disorders have been maintained constant, or in some disorders, increased more recently even after discounting diagnostic criteria changes in the diagnostic manuals (Torrey, 1987; King and Bearman, 2009; Visser et al., 2010).

Accumulating evidence suggests *de novo* mutations of the genes are a significant mechanism that causes psychiatric disorders such as schizophrenia and autism spectrum disorder, and could account for constant prevalence of these disorders (Sullivan et al., 2012; Veltman and Brunner, 2012). In contrast, it has been estimated that such *de novo* mutations could account for only a small percentage of cases, and a majority of cases is still caused by additive effects of multiple common genetic variants with each gene variant contributing a small effect (Mcclellan et al., 2007; Awadalla et al., 2010). Therefore, constant prevalence of psychiatric disorders cannot be fully explained by the mechanism of *de novo* mutations. Moreover, even if such *de novo* mutations were the major mechanism that causes psychiatric disorders, it is still unclear why specific neural systems such as the DA system have been chosen to be altered in these psychiatric disorders.

Indeed, behavioral traits associated with psychiatric disorders are thought to have emerged at some points of evolution, and have been inherited into humans. A number of hypotheses have been proposed, arguing that some aspects of psychiatric symptoms associated with DA alterations operate advantageously in specific environmental contexts. For instance, ADHD is one of such psychiatric disorders in which DA deficits have been implicated, but advantageous aspects of its symptoms are relatively clear. The core symptoms of ADHD consist of hyperactivity, impulsivity, and inattention, all of which involve DA signaling. Administration of DA agonists such as psychostimulants causes hyperlocomotion in rodents (Vanderschuren and Kalivas, 2000). Impulsivity can be examined using a behavioral task such as a delay discounting task in rodents in which a choice of either a large reward after a delay or a small reward without a delay is weighted. Using this test, administration of a D1 receptor antagonist has been shown to shift a choice for a more impulsive, immediate access to obtain a small reward over a delayed large reward (Koffarnus et al., 2011). An ability to focus and sustain attention to a particular target has also been shown to be impaired by administration of D1 and D2 antagonists in rodents (Pezze et al., 2007; Agnoli et al., 2013), non-human primates (Von Huben et al., 2006), and humans (Muller et al., 1998; Kahkonen et al., 2001; Mehta et al., 2004). These ADHD symptoms are inappropriate in the modern human social system. In contrast, they could yield great advantages for animals living in a wild environment and for humans (or ancestries of humans) living in a hunter-gatherer society (Jensen et al., 1997). Thus, exploration of a larger field and more frequent scanning of the field with a short span of attention as consequence of hyperactivity and impaired sustained attention, respectively, enable faster detection of a predator. In addition, impulsivity enables a quick decision to escape from a predator. These behavioral phenotypes could therefore facilitate survival and reproduction in a wild life environment.

Psychiatric disorders in which DA deficits are implicated exhibit cognitive dysfunction. Such cognitive deficits are often similar, if not identical, across different disorders. For instance, impulsivity is a behavioral trait that is observed not only in ADHD, but also in other psychiatric disorders such as schizophrenia (Ouzier, 2013), obsessive compulsive disorder (Fontenelle et al., 2011), and drug addiction (Fontenelle et al., 2011; Grant and Chamberlain, 2014). Similarly, an impairment of sustained attention is also not a unique feature of ADHD, but also observed in other psychiatric disorders in which DA deficits are implicated (Coull, 1998; Chen and Faraone, 2000; Bellgrove and Mattingley, 2008). Thus, some cognitive deficits associated with altered DA transmission may represent general and fundamental aspects of psychiatric conditions, and may have evolutionary overlapping roots in terms of adaptations to specific environmental conditions.

## Conclusions

In this article, we have presented the concept, along with supporting literatures, that alterations of the DA system associated with psychiatric disorders such as schizophrenia may not necessarily work disadvantageously in specific socioecological contexts of animals such as non-human primates. This may provide biological explanations why genetic variants on several psychiatric disorder-associated genes such as DRD2 and COMT have been selected or maintained through evolution in humans. Reconsideration of psychiatric disorders with DA deficits from an evolutionary perspective would yield novel insights into our understanding, and open a new venue on research unveiling the biological mechanisms, and thereby prevention and therapeutic treatments, of psychiatric disorders.

### Conflict of interest statement

The authors declare that the research was conducted in the absence of any commercial or financial relationships that could be construed as a potential conflict of interest.
